# Influence of adiposity and fatigue on the scapular muscle recruitment order

**DOI:** 10.7717/peerj.7175

**Published:** 2019-06-25

**Authors:** Guillermo Mendez-Rebolledo, Eduardo Guzman-Muñoz, Rodrigo Ramírez-Campillo, Pablo Valdés-Badilla, Carlos Cruz-Montecinos, Juan Morales-Verdugo, Francisco Jose Berral de la Rosa

**Affiliations:** 1 Escuela de Kinesiología, Facultad de Salud, Universidad Santo Tomás, Chile; 2 Universidad Pablo de Olavide, Seville, Spain; 3 Laboratory of Human Performance, Quality of Life and Wellness Research Group, Department of Physical Activity Sciences, Universidad de Los Lagos, Osorno, Chile; 4 Institute of Physical Activity and Health, Universidad Autónoma de Chile, Temuco, Chile; 5 Department of Physical Therapy, Laboratory of Clinical Biomechanics, Faculty of Medicine, University of Chile, Santiago, Chile

**Keywords:** Nutritional status, Anthropometry, Motor control, Muscle pattern, Timing

## Abstract

**Background:**

Several authors have indicated that excess body weight can modify the electromyographic (EMG) amplitude due to the accumulation of subcutaneous fat. This accumulation of adipose tissue around the muscle would affect the metabolic capacity during functional activities. On the other hand, some authors have not observed differences in the myoelectric manifestations of fatigue between normal weight and obese people. Furthermore, these manifestations have not been investigated regarding EMG onset latency, which indicates a pattern of muscle activation between different muscles. The objective of this study was to determine whether an increase in body weight, skinfolds, and muscle fatigue modify the trapezius and serratus anterior (SA) onset latencies and to determine the scapular muscle recruitment order in fatigue and excess body weight conditions.

**Methods:**

This cross-sectional study was carried out in a university laboratory. The participants were randomly assigned to the no-fatigue group (17 participants) or the fatigue (17 participants) group. The body mass index, skinfold thickness (axillary, pectoral, and subscapular), and percentage of body fat were measured. In addition, the onset latency of the scapular muscles [lower trapezius (LT), middle trapezius (MT), upper trapezius (UT), and SA] was assessed by surface EMG during the performance of a voluntary arm raise task. A multiple linear regression model was adjusted and analyzed for the additive combination of the variables, percentage body fat, skinfold thickness, and fatigue. The differences in onset latency between the scapular muscles were analyzed using a three-way repeated measure analysis of variance. In all the tests, an alpha level <0.05 was considered statistically significant.

**Results:**

For the MT, LT, and SA onset latencies, the body mass index was associated with a delayed onset latency when it was adjusted for the additive combination of percentage of body fat, skinfold thickness, and fatigue. Of these adjustment factors, the subscapular skinfold thickness (*R*^2^ = 0.51; *β* = 10.7; *p* = 0.001) and fatigue (*R*^2^ = 0.86; *β* = 95.4; *p* = 0.001) primarily contributed to the increase in SA onset latency. A significant muscle ×body mass index ×fatigue interaction (*F* = 4.182; *p* = 0.008) was observed. In the fatigue/excess body weight condition, the UT was activated significantly earlier than the other three scapular muscles (*p* < 0.001) and SA activation was significantly delayed compared to LT (*p* < 0.001).

**Discussion:**

Excess body weight, adjusted for skinfold thickness (axillary and subscapular) and fatigue, increases the onset latency of the MT, LT, and SA muscles and modifies the recruitment order of scapular muscles. In fact, the scapular stabilizing muscles (MT, LT, and SA) increase their onset latency in comparison to the UT muscle. These results were not observed when excess body weight was considered as an individual variable or when adjusted by the percentage body fat.

## Introduction

Overweight and obesity are defined as abnormal and excessive fat accumulation that is manifested by excess body weight and may impair health (World Health Organization, 2018). Overweight and obesity have been cataloged as a global pandemic that has caused worldwide concern due to the sustained increase in its prevalence (8%) between 1980 and 2013, mainly in children and young adults (Ng et al., 2014; Swinburn et al., 2011).

Cardiovascular (Ghoorah et al., 2016), biomechanical, neurophysiological (Lee et al., 2016), and metabolic (Garcia-Vicencio et al., 2015) alterations are among the negative consequences of excess body weight. In young adults, overweight and obesity lead to a decrease in force control and an increase in fatigue (Mehta, 2015; Mehta & Shortz, 2014). In addition, an alteration in the activation pattern of the scapular stabilizing muscles, in non-obese subjects has been observed, as reflected in the delayed activation of the scapular muscles under fatigue conditions (Cools et al., 2002; Mendez-Rebolledo et al., 2018a). However, there are no reports regarding the effect of excess body weight on the muscle activation pattern.

To assess neuromuscular function, researchers and clinicians often apply electromyography (EMG) (Hug, 2011; Struyf et al., 2014). Neuromuscular parameters that are frequently studied are signal amplitude, conduction velocity, fatigability, frequencies, and onset latency (Hug, 2011; Struyf et al., 2014). In the scapular muscles, the onset latency corresponds to the time between the EMG activation of a scapular muscle and the activation of the anterior deltoid (primary motor muscle) (Mendez-Rebolledo et al., 2018a; Phadke & Ludewig, 2013), which determines the muscle recruitment order during voluntary motor tasks. Several authors have indicated that excess body weight can reduce the EMG amplitude due to the accumulation of fat in the layers of tissue that separate the muscle from the skin (Cooper et al., 2014; De Vito et al., 2003; Phadke & Ludewig, 2013). The subcutaneous fat serves as a low-pass filter of the EMG, and therefore, would reduce the amplitude. Cooper et al. (2014), for instance, demonstrated that EMG M-waves were negatively correlated with skinfold tissue. Previous authors have suggested that the accumulation of adipose tissue around the muscle would affect the metabolic capacity and accelerates the appearance of muscle fatigue during functional activities (Garcia-Vicencio et al., 2015). On the other hand, some authors have not observed differences in the myoelectric manifestations of fatigue between normal weight and obese people (Minetto et al., 2013). Furthermore, these manifestations have not been investigated regarding EMG onset latency, which is considered one of the components that explains neuromuscular control (Cools et al., 2002; Phadke & Ludewig, 2013; Struyf et al., 2014). In this context, the effect of fatigue on the onset latency is known, but the combined effect of excess body weight and fatigue on the onset latency and scapular muscle recruitment order is unknown.

For this reason, it is of interest to determine whether an increase in body weight, skinfolds, and muscle fatigue modify the trapezius and serratus anterior (SA) onset latencies and to determine the scapular muscle recruitment order in fatigue and excess body weight conditions. We hypothesized that an increase in adiposity and fatigue in young adults with excess weight would modify the onset latency of the scapular muscles and scapular muscle recruitment order during a voluntary arm raise task. It is possible that the accumulation of fat around the muscle affects the normal mechanisms of fatigue development, such as an alteration in sarcolemma function, increase in the motor unit firing frequency, and decrease in the conduction velocity (Candotti et al., 2009; Dimitrova & Dimitrov, 2003). Therefore, this myoelectric alteration would modify the latency and recruitment order of the scapular muscles.

## Materials & Methods

### Design

The present investigation consisted of a cross-sectional study and was conducted in the Biomechanics and Motor Control Laboratory of the Universidad Santo Tomás (Talca, Chile). This investigation was designed considering the Helsinki Consensus (1975) on biomedical research in humans. The Ethic Scientific Committee of the Universidad Santo Tomás (Chile) approved all procedures (Folio ID-106) and an informed consent form was read and signed by each participant.

### Participants

The participants were selected through a non-probabilistic sample of students from the Health Faculty of the Universidad Santo Tomás (Talca, Chile) recruited via advertising. A total sample of 34 voluntary participants (17 participants per group: no-fatigue and fatigue) was calculated based on a 95% confidence interval, a power of 0.9, and an expected 15% loss. For this calculation, SA onset latency reported in a previous study was used (Mendez-Rebolledo et al., 2018a), where a mean difference of 53.3 ms and a standard deviation of 38.4 ms was observed in the no-fatigue condition and 55.1 ms was observed in the fatigue condition. Participants between 18 and 24 years of age were included. Exclusion criteria were: (1) incomplete range of motion of the shoulder; (2) history of trauma, dislocation, rotator cuff tear, spinal deformities, radicular symptoms, and/or neurological diseases; (3) participation in overhead sports (e.g., handball); (4) presence of scapular dyskinesis; (5) a current or past history of shoulder pain.

### Instrumentation

The body weight was assessed with a scale (Seca, Hamburg, Germany; 0.1 kg accuracy), standing height was measured using a stadiometer (Seca, model 220, USA; 0.1 cm accuracy), and the pectoral, axillary, triceps, subscapular, suprailiac, abdominal, and anterior thigh skinfolds were measured with a Lange caliper Model C-130 (precision 0.5 mm) (Creative Health Products, Inc., Ann Arbor, MI, USA). The surface EMG and acceleration signals were acquired with a Delsys Trigno™ Wireless sEMG System and recorded with the Delsys EMGworks Acquisition 4.2.0 (Delsys Inc., Boston, MA, USA). Two different sensors were used on the shoulder, one sensor for surface EMG and another as an accelerometer. The beginning and end of the arm raise task (abduction-adduction cycle) was determined with an accelerometer (Delsys Inc. Boston, MA, USA) on the anterior deltoid surface of the dominant arm (Mendez-Rebolledo et al., 2016). The accelerometer was used to control the abduction-adduction cycle and was not used as a reference to determine electromechanical delay. To select the dominant upper limb, a reach-to-grasp task was used, which consisted of reaching and grasping an object positioned in the ipsilateral, middle, and contralateral spaces. The hand that reached the target in the ipsilateral and middle spaces was selected as the dominant upper limb. The surface EMG electrodes were made of 99% silver and had an inter-electrode distance of 10 mm. The surface EMG was sampled at 2,000 Hz, amplified with a gain of 300, and filtered with a bandpass filter (fourth-order, Butterworth filter with frequencies between 20 and 450 Hz).

### Procedures

All procedures were performed in one session. Body weight, height, body mass index (BMI), and cutaneous skinfold thickness were assessed. The participants were classified in relation to nutritional status according to the World Health Organization statements: normal weight (BMI 18.5 to 24.9 kg/m2), overweight (BMI 25.0 to 29.9 kg/m2) and obese (BMI ≥ 30 kg/m2). The skinfolds were measured according to a protocol described by the International Society for Advances in Kinanthropometry (ISAK) (Marfell-Jones et al., 2012). The skinfolds of both upper limbs were measured to determine possible differences between sides. The skinfold site was carefully located using the correct anatomical landmarks: For pectoral, the skinfold was raised at a point between the axilla and nipple as high as possible on the anterior axillary fold; for axillary, the skinfold was raised at the point where a vertical line from the mid axilla intersects with a horizontal line level with the bottom edge of the xiphoid process; for triceps, the skinfold was raised at the level of the mid-point between the acromion and the head of radius, on the midline of the posterior surface of the arm; for subscapular, the line of the skinfold was determined by the natural fold lines of the skin; for suprailiac, the line of the skinfold ran slightly downward, posterior-anterior, as determined by the natural fold lines of the skin; for abdominal, the fold was parallel to the navel; for anterior thigh, the skinfold was raised at the mid-point of the anterior surface of the thigh, midway between patella and inguinal fold. The skinfolds were picked up at the marked line. The skinfolds were grasped and lifted so that a double fold of skin plus the underlying subcutaneous adipose tissue was held between the thumb and index finger of the left hand. The near edge of the thumb and finger were in line with the marked site. The nearest edge of the contact faces of the caliper were applied 1 cm away from the edge of the thumb and finger. The caliper was held at 90° to the surface of the skinfold site at all times. Two certified evaluators with ISAK level II made the measurements. Each skinfold was measured twice by an evaluator (technical measurement error 0.91%) and a third measurement was made by a different evaluator (technical measurement error 0.89%). Then, the median of the three repetitions performed was selected. The percentage of body fat (%BF) was obtained by the Siri equation, where the body density considered was the one proposed in the Jackson & Pollock equation (Jackson & Pollock, 1978):

Siri equation:

\begin{eqnarray*}\text{%}BF= \left( \frac{495}{\text{Body} \text{Density}} \right) -450 \end{eqnarray*}

Jackson & Pollock equation:

\begin{eqnarray*}\text{Body}~\text{Density}=1.112- \left( 0.00043499\times \sum \text{skin}~\text{folds} \right) + \left( 0.00000055\times \sum \text{skin}~{\text{folds}}^{2} \right) \nonumber\\\displaystyle - \left( 0.00028826\times \text{age} \right) \end{eqnarray*}

Where the skinfolds (measured in mm) are: axillary, pectoral, tricipital, subscapular, abdominal, suprailiac and anterior thigh.

The participants were randomly assigned (random number generator) to the no-fatigue (17 participants) group or the fatigue (17 participants) group. Then, the location of the electrodes in the anterior deltoid, upper trapezius (UT), middle trapezius (MT), lower trapezius (LT), and SA muscles was prepared on the dominant arm. This included shaving the hair, then cleaning with dermabrasive paper and 70% isopropyl alcohol. The electrodes were placed longitudinally to the fibers of the muscles according to previous recommendations (Hermens et al., 2000): The electrode for UT was placed at 50% on the line from the acromion to the spine on vertebra C7, the electrode for MT was placed at 50% between the medial border of the scapula and the spine, at the level of T3, and the electrode for LT was placed at 2/3 on the line from the trigonum spinea to the 8th thoracic vertebra. In the case of the SA, the electrode was located according to a previous study (Lehman, Gilas & Patel, 2008), thus, the electrode was placed on the muscle belly in the mid-axillary line over the fifth rib. Details on the placement of the electrodes are described in a recently published report by Mendez-Rebolledo et al. (2018a).

At the beginning of the session, warm-up exercises consisting of elongations of the glenohumeral (internal and external rotators) and scapular muscles (UT, pectoralis minor, and scapular elevator) were performed. For all elongations, five repetitions of 15 s were used. The no-fatigue and fatigue groups performed a voluntary arm raise task, which consisted of nine abduction-adduction cycles that took less than a minute, consisting of a dominant arm elevation in the scapular plane with a velocity of 4 s per cycle of abduction and adduction (Sugamoto et al., 2002). Before the task, the participants were instructed to reproduce the movement velocity following the established rhythm of a metronome and practiced the movement ten times with the aim of allowing a familiarization of the task. The movement of the arm was executed voluntarily, without interruptions, and in the presence of proprioceptive and visual information in order to consider the movement as a “predictable perturbation” (Aruin, Shiratori & Latash, 2001). In this investigation, keeping the eyes open and the joints without restrictions (orthosis, limited ranges, clothing) corresponded to making a movement in the presence of visual and proprioceptive information.

### Fatigue protocol

The fatigue group performed a cycle of abduction and adduction of the dominant arm in the scapular plane with a velocity of 1 s per cycle, as many times as possible. The movement was performed with a dumbbell according to body weight, 1.4 kg for those participants with a weight less than 68.1 kg and 2.3 kg for those with a body weight greater than 68.1 kg (McClure et al., 2009). Before initiating the fatigue protocol, participants received instructions on the Modified Borg Effort Perception Scale (Borg, 1990). Participants were asked questions regarding the level of fatigue of the shoulder, on a scale of 0 to 10, after every 20 cycles of abduction and adduction. The fatigue protocol was interrupted when the participants reached a score equal to or greater than 8 (Zanca, Grüninger & Mattiello, 2016) and were not able to maintain the arm elevation. The time of task failure was 160 ± 14 s. Finally, the participants again performed the voluntary arm raise task according to the procedure described above.

### Data processing

The signals were full-wave rectified and filtered with a low-pass filter (50 Hz, fourth-order, Butterworth filter) (Phadke & Ludewig, 2013). The surface EMG onset latency was calculated through a visual inspection method based on the average and standard deviation of the resting surface EMG signal. The average and standard deviation were calculated in relation to a period of 200 ms of rest signal prior to the initiation of the arm raise task. The three central cycles of abduction-adduction were obtained. In each cycle, the average and standard deviation of the resting surface EMG signal were calculated and the signal that presented the median was selected. Then, the onset was defined as the point where the EMG activity passed the threshold of at least three standard deviations above the average of the signal at rest and maintained this level of activation for at least 25 ms (Mendez-Rebolledo et al., 2018b; Phadke & Ludewig, 2013). Finally, onset latency for each scapular muscle was calculated as the difference in latency relative to that of primary motor muscle of the arm flexion (Mendez-Rebolledo et al., 2018a; Mendez-Rebolledo et al., 2016; Phadke & Ludewig, 2013), i.e., anterior deltoid. Two researchers processed the signal, one calculated the onset latency and another corroborated it. The procedure was repeated until agreement was reached. If an artifact was found, the signal was removed. All raw EMGs signals were analyzed with EMGworks Analysis 4.2.0 (Delsys Inc. Boston, MA, USA).

### Statistical analysis

The software SPSS 22.0 was used to perform the statistical analysis of the data. In all tests, an alpha level <0.05 was considered. The mean and standard deviation were calculated to describe the baseline characteristics of the sample: age, body weight, height, BMI, skinfold thickness, %BF, and onset latency of the scapular muscles. The distributions of normality, homogeneity of variance, and sphericity were evaluated with the Shapiro–Wilk test, Levene’s test, and Mauchly’s sphericity test, respectively. In addition, one-way analysis of variance (ANOVA) was performed to determine differences in the basal characteristics of the sample, and a dependent *t*-test was used to compare skinfold thickness measurements between the dominant and non-dominant arms. The following statistical analyses were carried out: (1) the relationship between BMI, %BF, and skinfold thickness with onset latency for each scapular muscle was analyzed using Pearson’s test. A correlation coefficient r from 0 to 0.4 was considered as weak, 0.41 to 0.7 as moderate, and 0.71 to 1.0 as strong (Cohen, 1988); (2) the influence of the BMI on the onset latency of each scapular muscle was analyzed through a multiple linear regression model adjusted for the additive combination of the variables: %BF, skinfolds (axillary, pectoral and subscapular), and fatigue; (3) the differences in onset latency between the scapular muscles were analyzed through three-way repeated measures, using ANOVA, within and between the following factors: muscle (four levels), BMI (two levels), and fatigue (two levels). Bonferroni corrected t-tests were used to compare the onset latencies between muscles. The muscle recruitment order was identified through the average onset latency of each scapular muscle and group. For these last two statistical analyses, the BMI was analyzed as a dichotomous variable, normal weight and excess body weight (sum of overweight and obese).

## Results

All participants were included in the analysis, as no participants presented EMG signals with excessive noise and artifacts. Therefore, the following results included 17 participants for each group (no-fatigue and fatigue). All data presented a normal distribution and homogeneity of variance. Table 1 shows the basal characteristics of the sample. There were no significant differences in BMI and skinfold thickness between the fatigue and no-fatigue groups (*p* > 0.05).

**Table 1:** Basal characteristics of the sample.

	**Group non-fatigue(*n* = 17)**		**Group fatigue(*n* = 17)**		
	**NW (*n* = 9)**	**O/O (*n* = 8)**	**NW (*n* = 7)**	**O/O (*n* = 10)**	
	**(Mean ± SD)**	**(Mean ± SD)**	**(Mean ± SD)**	**(Mean ± SD)**	***P***
**Age (years)**	21.4	23.0	21.7	23.4	0.610
**Body weight (kg)**	74.2	78.8	66.0	84.5	0.808
**Height (m)**	1.77	1.70	1.73	1.75	0.808
**Body mass index (kg/m**^**2**^**)**	23.46	27.28	22.07	27.32	0.408
**Body fat (%)**	16.8 ± 2.4	19.5 ± 1.8	15.1 ± 2.7	20.6 ± 1.6	0.077
**Axillary skinfold (mm)**	14.3 ± 2.2	17.2 ± 2.5	13.2 ± 2.7	20.7 ± 1.6	–
**Pectoral skinfold (mm)**	13.3 ± 3.7	16.0 ± 3.9	10.8 ± 3.0	14.9 ± 3.3	–
**Subscapular skinfold (mm)**	21.1 ± 2.3	22.6 ± 2.0	20.0 ± 1.9	26.8 ± 3.1	–
**UT onset latency (ms)**	−28.5 ± 44.3	−43.5 ± 23.8	−32.8 ± 56.9	−59.6 ± 26.9	–
**MT onset latency (ms)**	−61.8 ± 28.0	−105.0 ± 69.1	15.8 ± 47.1	54.6 ± 25.5	–
**LT onset latency (ms)**	−74.6 ± 46.1	−59.2 ± 44.2	7.5 ± 28.5	36.8 ± 17.6	–
**SA onset latency (ms)**	−73.6 ± 47.6	−73.4 ± 35.4	−3.8 ± 20.4	80.6 ± 24.3	–

**Notes.**

NW normoweight O/O overweight/obesity UT upper trapezius MT middle trapezius LT lower trapezius SA serratus anterior SD standard deviation

*P*: statistical significance between non-fatigue and fatigue groups.

Tables 2 and 3 show the correlations between scapular muscles onset latencies and BMI, skinfolds, %BF, and fatigue. Fatigue showed a significantly moderate or strong correlation with the MT, LT, and SA onset latency. In addition, the subscapular skinfold thickness was the only one that showed a significant moderate correlation with the SA onset latency (*R*^2^ = 0.51).

**Table 2:** Multiple linear regression model of the onset latency of upper trapezius and middle trapezius, adjusted by the additive combination of the variables: body mass index, percentage of body fat, skinfolds, and fatigue in no-fatigue (*n* = 17) and fatigue (*n* = 17) groups.

		***R***^**2**^	*β*	***P***	**95% CI**	
**Upper Trapezius Onset Latency**	**Model 1 - Body Mass Index**	0.08	−37.6	0.071	−78.7	3.4
	**Model 2 - Body Fat percentage**	0.10	1.0	0.796	−7.3	9.4
	**Model 3 - Axillary Skinfold**	0.11	2.4	0.517	−5.1	9.9
	**Model 4 - Fatigue**	0.13	−13.6	0.357	−43.5	16.2
	**Model 1 - Body Mass Index**	0.08	−32.6	0.089	−70.6	5.3
	**Model 2 - Body Fat percentage**	0.10	3.6	0.318	−3.7	11.1
	**Model 3 - Pectoral Skinfold**	0.10	−1.1	0.661	−6.1	3.9
	**Model 4 - Fatigue**	0.13	−12.2	0.405	−41.8	17.3
	**Model 1 - Body Mass Index**	0.08	−31.2	0.112	−70.1	7.7
	**Model 2 - Body Fat percentage**	0.10	3.4	0.356	−4.0	10.9
	**Model 3 - Subscapular Skinfold**	0.11	−0.8	0.767	−6.8	5.1
	**Model 4 - Fatigue**	0.12	−8.3	0.573	−38.1	21.5
**Middle Trapezius Onset Latency**	**Model 1 - Body Mass Index**	0.00	−36.7	0.130	−85.1	11.5
	**Model 2 - Body Fat percentage**	0.00	−3.7	0.441	−13.6	6.0
	**Model 3 - Axillary Skinfold**	0.27	9.5	0.035^*^	0.7	18.4
	**Model 4 - Fatigue**	0.69	107.0	0.000^*^	71.9	142.1
	**Model 1 - Body Mass Index**	0.00	−15.9	0.502	−63.7	31.9
	**Model 2 - Body Fat percentage**	0.00	2.0	0.658	−7.3	11.4
	**Model 3 - Pectoral Skinfold**	0.06	1.6	0.610	−4.8	8.0
	**Model 4 - Fatigue**	0.64	125.1	0.000^*^	87.8	162.5
	**Model 1 - Body Mass Index**	0.00	−22.0	0.357	−70.2	26.1
	**Model 2 - Body Fatpercentage**	0.00	0.5	0.904	−8.7	9.8
	**Model 3 - Subscapular Skinfold**	0.17	4.1	0.265	−3.3	11.5
	**Model 4 - Fatigue**	0.65	114.1	0.000^*^	77.2	151.0

**Notes.**

* significant difference (*P* < 0.05); 95% CI, 95% confidence interval.

The multiple linear regression model showed that UT onset latency was not significantly associated with the BMI, skinfold thickness, %BF, or fatigue (*p* > 0.05) (Table 2). For the MT and LT onset latencies, the BMI was associated with a delayed muscle onset latency when it was adjusted by model 3 (additive combination of %BF and axillary skinfold thickness) (Table 2) and model 4 (additive combination of BF%, axillary skinfold thickness, and fatigue) (Table 3). For the SA onset latency, the multiple linear regression model showed that BMI was associated with a delayed onset latency when it was adjusted for models 2, 3 (axillary and subscapular skinfold thickness), and 4 (Table 3). Of these adjustment factors, subscapular skinfold thickness (*R*^2^ = 0.51; *β* = 10.7; *p* = 0.001) and fatigue (*R*^2^ = 0.86; *β* = 95.4; *p* = 0.001) contributed most to the increase in SA onset latency.

**Table 3:** Multiple linear regression model of the onset latency of lower trapezius and serratus anterior, adjusted by the additive combination of the variables: body mass index, percentage of body fat, skinfolds, and fatigue in no-fatigue (*n* = 17) and fatigue (*n* = 17) groups.

		***R***^**2**^	*β*	***P***	**95% CI**	
**Lower Trapezius Onset Latency**	**Model 1 - Body Mass Index**	0.08	−15.6	0.336	−48.4	17.0
	**Model 2 - Body Fat percentage**	0.10	−0.1	0.968	−6.8	6.5
	**Model 3 - Axillary Skinfold**	0.37	7.4	0.016^*^	1.4	13.4
	**Model 4 - Fatigue**	0.75	79.2	0.000^*^	55.4	103.0
	**Model 1 - Body Mass Index**	0.08	0.2	0.987	−33.0	33.5
	**Model 2 - Body Fat percentage**	0.10	5.8	0.077	−0.6	12.3
	**Model 3 - Pectoral Skinfold**	0.19	−0.6	0.782	−5.0	3.8
	**Model 4 - Fatigue**	0.70	89.4	0.000^*^	63.4	115.4
	**Model 1 - Body Mass Index**	0.08	−2.1	0.899	−35.9	31.7
	**Model 2 - Body Fatpercentage**	0.10	4.2	0.196	−2.3	10.7
	**Model 3 - Subscapular Skinfold**	0.23	1.7	0.499	−3.4	6.9
	**Model 4 - Fatigue**	0.70	87.5	0.000^*^	61.5	113.4
**Serratus Anterior Onset Latency**	**Model 1 - Body Mass Index**	0.14	6.4	0.738	−32.7	45.6
	**Model 2 - Body Fat percentage**	0.15	−0.5	0.885	−8.5	7.4
	**Model 3 - Axillary Skinfold**	0.36	7.2	0.047^*^	0.1	14.4
	**Model 4 - Fatigue**	0.78	104.2	0.000^*^	75.7	132.7
	**Model 1 - Body Mass Index**	0.14	21.3	0.243	−15.3	58.1
	**Model 2 - Body Fat percentage**	0.15	8.0	0.030^*^	0.8	15.2
	**Model 3 - Pectoral Skinfold**	0.33	−4.2	0.089	−9.1	0.6
	**Model 4 - Fatigue**	0.77	106.5	0.000^*^	77.9	135.2
	**Model 1 - Body Mass Index**	0.14	6.7	0.639	−22.5	36.1
	**Model 2 - Body Fat percentage**	0.15	−2.3	0.401	−7.9	3.2
	**Model 3 - Subscapular Skinfold**	0.51	10.7	0.000^*^	6.2	15.3
	**Model 4 - Fatigue**	0.86	95.4	0.000^*^	72.9	117.8

**Notes.**

* significant difference (*P* < 0.05); 95% CI, 95% confidence interval.

**Figure 1:**
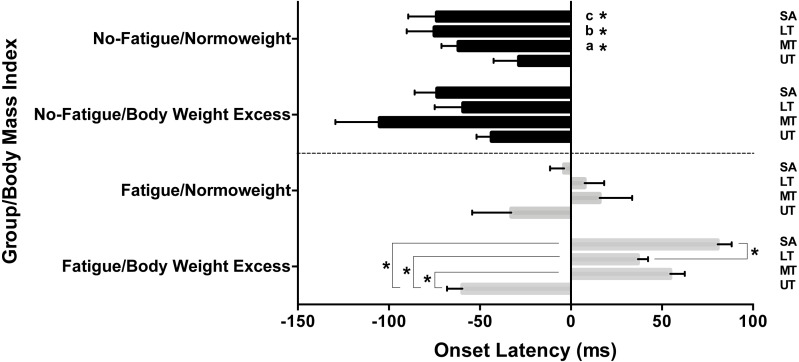
Scapular muscles onset latencies and recruitment order of two groups (fatigue and no-fatigue) and two body mass indexes [normoweight and body mass excess (overweight and obese)]. SA, serratus anterior; LT, lower trapezius; MT, middle trapezius; UT, upper trapezius. ^a^MT in No-Fatigue/Normoweight condition was activated significantly earlier than in Fatigue/Normoweight and Fatigue/Body Weight Excess conditions. ^b^LT in No-Fatigue/Normoweight condition was activated significantly earlier than in Fatigue/Normoweight and Fatigue/Body Weight Excess conditions. ^c^SA in No-Fatigue/Normoweight condition was activated significantly earlier than in Fatigue/Normoweight and Fatigue/Body Weight Excess conditions.*P < 0.001.

The repeated measures ANOVA revealed a significant muscle ×BMI ×fatigue interaction (*F* = 4.182; *p* = 0.008). The post-hoc analysis showed that MT, LT, and SA were activated significantly earlier in the no-fatigue/normal weight condition than the same muscles in the fatigue/normal weight (*p* < 0.01) and fatigue/excess body weight conditions (*p* < 0.01) (Fig. 1). The no-fatigue/normal weight, no-fatigue/excess body weight, fatigue/normal weight conditions did not show significant differences between the scapular muscles. In general, the muscle recruitment order was LT, MT (or MT, LT), SA, and UT. Conversely, in the fatigue/excess body weight condition, the UT was activated significantly earlier than the other three scapular muscles (*p* < 0.001). In addition, the SA was activated significantly later than LT (*p* < 0.001) (Fig. 1). In the latter situation, the muscle recruitment order was: UT, LT, MT, and SA.

## Discussion

The main results of the present study indicate that excess body weight, adjusted for skinfold thickness (axillary and subscapular) and fatigue, increases the onset latency of the MT, LT, and SA muscles and modifies the recruitment order of the scapular muscles. In fact, the scapular stabilizing muscles (MT, LT, and SA) increased (delay) their onset latency in comparison to the UT muscle. These results were not observed when excess body weight was considered as an individual variable or when it was adjusted by the %BF. The contribution of these variables to the delayed latency of the scapular muscles was between 7.2–10.7 ms (axillary and subscapular skinfold thickness) and 79.2–125.1 ms (fatigue). To the best of our knowledge, this is the first investigation to determine the simultaneous effect of excess body weight, skinfold thickness, and fatigue on the onset latency of scapular muscles.

Obesity can alter the normal mechanisms of fatigue development due to the associated physiological and neuromuscular changes (Pajoutan, Ghesmaty Sangachin & Cavuoto, 2017). During sustained submaximal contractions, both peripheral and central changes lead to myoelectric manifestations undergoing fatigue prior to task failure (Enoka & Duchateau, 2008; Gandevia, 2001). These myoelectric manifestations correspond to an increase in the motor units firing frequencies, EMG amplitude, onset latency, and a decrease in the conduction velocity (Adam & De Luca, 2003; Candotti et al., 2009; Cools et al., 2002; Mendez-Rebolledo et al., 2018a). Several authors have hypothesized that these myoelectric manifestations are a response of the central nervous system to the electrochemical imbalance in the muscle fiber and the reduction of the propagation velocity of intracellular action potential (Allen & Westerblad, 2001; Dimitrova & Dimitrov, 2003; Sjøgaard, 1996). In this sense, the myoelectric manifestations of fatigue would be enhanced or exacerbated in the presence of intramuscular and subcutaneous fat, since some reports have shown a significant relationship between fat and the expression of pro-inflammatory cytokines in the muscle (Addison et al., 2014; Coppack, 2001; Mohamed-Ali et al., 1997), which could alter the electrochemical balance and neural conductivity. This alteration is one of the main causes of the changes occurring in amplitude and spectral EMG variables during fatigue (Dimitrova & Dimitrov, 2003). Therefore, it is reasonable to think that if the EMG amplitude and spectrum are modified by fatigue –and more the enhancing effect of intramuscular and subcutaneous fat–the onset latency could also be modified. However, this explanation should be considered with caution since it is necessary to carry out future investigations that clarify the enhancing effect of intramuscular and subcutaneous fat in the myoelectric manifestations of a fatigued muscle.

Previous reports have not observed differences in the strength of elbow flexion (Mehta & Shortz, 2014) and muscular resistance (De Vito et al., 2003; Minetto et al., 2013) between normal weight and obese people. It is possible that no differences are observed in these variables because the motor task is not demanding enough for neuromuscular control. The latter is determined by a wide range of neural (motor unit firing frequencies, maximum voluntary force, conduction velocity, etc.) and contractile (fiber type, muscle–tendon unit stiffness, etc.) factors (Blazevich et al., 2009; Folland, Buckthorpe & Hannah, 2014). Of these factors, the decrease in the firing frequency and the conduction velocity of the motor units are the main mechanisms that may explain the development of fatigue (Adam & De Luca, 2003; Candotti et al., 2009) and possibly explain the increase in onset latency of fatigued muscles (Cools et al., 2002). For the other hand, it has been observed that obese people increase the recruitment of motor units, a higher neural cost, to prolong a muscular resistance task previous to the fatigue (Duan et al., 2018) and decrease mean power frequency at the fatigue threshold of the quadriceps muscle during a cycle ergometer task (Baniqued et al., 2016). In this context, it is possible that people with excess weight (increase in %BF, BMI and skinfolds) and fatigued muscles have an increase in onset latency due to the decrease in the firing frequency and conduction velocity of the motor units, both mechanisms observed in subjects with excess weight and muscle fatigue. In addition, the current study indicates that higher skinfold thickness and fatigue increase the onset latency of the SA and therefore modify the recruitment order of the scapular stabilizing muscles. Conversely, in normal weight and non-fatigued individuals the muscle activation was: MT, LT, SA, and UT. This result is similar to that observed by other authors (Kibler et al., 2007; Mendez-Rebolledo et al., 2018a). Mendez-Rebolledo et al. (2018a) evaluated the order of recruitment of non-fatigued scapular muscles in normal weight subjects during an arm raise task. They observed an early activation of the scapular stabilizing muscles (MT, LT, SA), followed by the anterior deltoid, and finally the UT. In the same way, Kibler et al. (2007) identified a similar recruitment order in healthy tennis players during a service. They observed an early activation of the SA, followed by the anterior deltoid, UT and other shoulder muscles (Kibler et al., 2007). This early activation of the scapular muscles during arm raise allows dynamic control of the scapula, followed by the positioning of the arm by the deltoid and finally the stabilization of the humeral head by the rotator cuff (Hirashima et al., 2002; Kibler et al., 2007). This proximal to distal recruitment pattern allows efficient muscle activation, which optimizes the production of force along the joints of the upper limb (Hirashima et al., 2002). In fact, this proximal to distal pattern has been observed in other body regions, for example, in the lower limb where early activation of the spine and hip muscles followed by the muscles of the knee and leg has been observed, which causes an increase in the ground reaction force and height of a countermovement jump (Mendez-Rebolledo et al., 2018a). In this sense, some authors have proposed through computational models that the performance of a motor task depends on an adequate muscle recruitment order and force production (Bobbert & Van Soest, 1994; Prokopow, Szyniszewski & Pomorski, 2005). Therefore, it is presumed that individuals with excess fat and fatigue would have an inefficient motor performance reflected, for example, in the production of force.

There are some limiting factors that reduced the generalizability of the findings in the current study. First, the age and sex recruitment criteria in this study were limited to younger males. In addition, the analyses of the EMG results revealed considerable intra- and inter-individual variation. One reason for this variation is the difference in the thickness and electrical properties of the adipose tissue layers between the surface electrodes and the muscle (Nordander et al., 2003). The distribution and thickness of subcutaneous fat is non-uniform along the muscle, which can affect the conduction velocity of different motor units that make up a muscle (Cescon, Rebecchi & Merletti, 2008), and therefore, have an impact on the estimation of muscle onset latency. In addition, the subcutaneous tissue thickness has an inverse relationship with EMG amplitude, as demonstrated in the literature (Cooper et al., 2014; De Vito et al., 2003; Nordander et al., 2003; Petrofsky, 2008). Once the muscle onset latency was estimated from EMG, differences in subcutaneous tissue could act as a non-physiological factor, i.e., a low-pass filter that affects the muscle onset latency estimation (Petrofsky, 2008). In order to reduce this limitation, the present study included participants with a similar BMI, %BF, and skinfold thickness in each group (fatigue and no-fatigue). However, the most reliable way to measure intramuscular and superficial adipose tissue is through ultrasound or magnetic resonance imaging (Young et al., 2015). These are costly and less extrapolatable techniques in the clinic, where it is necessary to quickly measure the skinfold thickness prior to an EMG evaluation. Another limitation of the present study was that the skinfolds were not measured under the EMG electrodes. Although the influence of subcutaneous fat is known as a low-pass filter that attenuates the surface EMG signal, it was decided not to perform this procedure because ISAK (skinfold measurement) (Marfell-Jones et al., 2012) and ISEK (localization of surface EMG electrodes) (Hermens et al., 2000) provide topographic recommendations which define the local fat accumulation and the most representative localization of muscle electrical activity, respectively. Therefore, respecting these recommendations allows a reliable and representative record of electrical activity of a muscle and the local fat accumulation, and at the same time, it allows the %BF to be calculated. In line with this, the analysis of the influence of obesity in a single procedure from a local and global point of view is facilitated. On the other hand, some references included in this manuscript (Minetto et al., 2013; Nordander et al., 2003) used ultrasound to measure the thickness of the skinfolds. These investigations did not respect the recommendations for ultrasound fat measurements (Stolk et al., 2001; Störchle et al., 2017), and therefore, could have a high variability and error in the estimation of subcutaneous fat.

## Conclusions

This study illustrated that excess body weight, added to the skinfold thickness (axillary and subscapular) and fatigue, increases the onset latency of the MT, LT, and SA muscles and modifies the recruitment order of the scapular muscles. These results should be considered during surface electromyography assessment procedures and in the performance of fatigued motor tasks.

##  Supplemental Information

10.7717/peerj.7175/supp-1 Supplemental Information 1 Suplementary raw data Raw data exported from the anthropometric and surface electromyographic evaluations applied for data analyses and preparation for Fig. 1, Tables 1–3.

## Additional Information and Declarations

### Data Availability

The following information was supplied regarding data availability: The raw measurements are available in File S1.
